# When Thermodynamic Properties of Adsorbed Films Depend on Size: Fundamental Theory and Case Study

**DOI:** 10.3390/nano10091691

**Published:** 2020-08-27

**Authors:** Bjørn A. Strøm, Jianying He, Dick Bedeaux, Signe Kjelstrup

**Affiliations:** 1 Department of Structural Engineering, Faculty of Engineering Science and Technology, Norwegian University of Science and Technology, NO-7491 Trondheim, Norway; jianying.he@ntnu.no; 2 Porelab, Department of Chemistry, Norwegian University of Science and Technology, NO-7491 Trondheim, Norway; dick.bedeaux@ntnu.no (D.B.); signe.kjelstrup@ntnu.no (S.K.)

**Keywords:** adsorption, thin film, nanothermodynamics, small-system, size-dependent, thermodynamics, spreading pressure, entropy of adsorption

## Abstract

Small system properties are known to depend on geometric variables in ways that are insignificant for macroscopic systems. Small system considerations are therefore usually added to the conventional description as needed. This paper presents a thermodynamic analysis of adsorbed films of any size in a systematic and general way within the framework of Hill’s nanothermodynamics. Hill showed how to deal with size and shape as variables in a systematic manner. By doing this, the common thermodynamic equations for adsorption are changed. We derived the governing thermodynamic relations characteristic of adsorption in small systems, and point out the important distinctions between these and the corresponding conventional relations for macroscopic systems. We present operational versions of the relations specialized for adsorption of gas on colloid particles, and we applied them to analyze molecular simulation data. As an illustration of their use, we report results for CO${}_{2}$ adsorbed on graphite spheres. We focus on the spreading pressure, and the entropy and enthalpy of adsorption, and show how the intensive properties are affected by the size of the surface, a feature specific to small systems. The subdivision potential of the film is presented for the first time, as a measure of the film’s smallness. For the system chosen, it contributes with a substantial part to the film enthalpy. This work can be considered an extension and application of the nanothermodynamic theory developed by Hill. It provides a foundation for future thermodynamic analyses of size- and shape-dependent adsorbed film systems, alternative to that presented by Gibbs.

## 1. Introduction

Adsorption is a central process in nature and in engineering. An important case in nature is adsorption on particles in the atmosphere. It has been recognized for many years that the representation of cloud processes is a significant source of uncertainty in climate models. Many interactions among particles in the atmosphere, clouds and precipitation are relevant for such processes and are the focus of a large area of climate change research [1]. The particles may vary in type, size and shape, and may originate from phenomena such as forest fires and volcanic eruptions, or from human activity such as industry and transportation. They follow the rising air through expansion and cooling, and serve, among other things, as condensation and ice nucleation sites. The initiation of condensation on particles begins with the adsorption or absorption of molecules from the surrounding air. Therefore, the development of more accurate climate models will benefit from a better understanding of these processes for small, curved particles. Although we use herein condensation on particles in the atmosphere as an important application, the focus of this work is on the fundamentals of adsorption on small systems in general.

A substantial amount of research from the 1930s onward on size-effects in thermodynamics may be found, for instance, in the literature on nucleation [2,3,4,5,6,7,8]. The majority of the work is based on Gibbs’ theory of heterogeneous systems [9]. However, there exists an alternative to the method of Gibbs, developed by Hill in the early 1960s [10]. Hill’s method distinguishes itself by the fact that it is a unified and systematic approach to the treatment of all small systems, and does not require the concept of dividing surfaces to be introduced at the outset. The equivalence of Hill’s and Gibbs’ method in its most general form for the description of curved surfaces has recently been verified [11]. The method is a generalization of macroscopic thermodynamics to small systems, and thus contains conventional thermodynamics as a special case in the macroscopic limit. By generalizing the fundamental differential equations traditionally used in thermodynamics, the whole internal structure of thermodynamic relations for small systems follows naturally by the familiar methods such as Euler integration, which otherwise would not apply for size dependent systems. The analogue of the Gibbs–Duhem equation for small systems is then readily available, and relations describing the size- and shape-dependence of all intensive properties follow. This has motivated us to describe adsorption on small systems using Hill’s theory to provide a solid foundation for the present and similar investigations. The aim of the study was thus to present a set of equations for adsorption on a small adsorbent, alternative to existing theories, most prominently Gibbs, and illustrate the set using molecular simulations of CO${}_{2}$ adsorption on a small sphere of graphite.

In conventional thermodynamics an adsorption system may be described in several ways, one of which considers the system to be the adsorbed phase only. The adsorbent properties are then subtracted from the properties of the surroundings, with the exception of the interaction energy with the adsorbed phase [12]. This view recognizes the asymmetric nature of the system and leads naturally to thermodynamic properties per film molecule. Another approach is to treat the system as a solution of adsorbent and adsorbate [13]. This application is referred to as solution thermodynamics. Though both approaches may be applied, the potential usefulness of one over the other relates to the possible interchange of adsorbent and adsorbate components. In the asymmetric approach, hereafter referred to as adsorption thermodynamics, the focus is on obtaining thermodynamic properties and relations for the adsorbed phase. In the systems typically considered, the properties are categorized as extensive or intensive depending on whether they are proportional to or independent of the size of the film. Therefore, even though the size is necessary in order to completely characterize the film, the nature of the film appears to be independent of it. As a consequence, the differential thermodynamic relations for the adsorbed phase are Euler homogeneous and of first degree in the extensive properties [14]. The relations are therefore directly integrable by the theorem of Euler.

For small systems, the statements above must be modified. The properties normally referred to as intensive are no longer independent of size, so the conventional use of the terms intensive and extensive must now refer to properties that obtain the implied characteristics in the macroscopic limit. The differential thermodynamic relations for a single system are no longer linear homogeneous functions, and can not be directly integrated as before. As the nature of the film now depends on its size and shape, it becomes of interest to systematically investigate the effects of size and shape in a framework that allows for this in a general way. This is possible in the thermodynamic theory of small systems, or nanothermodynamics, as developed by Terrell L. Hill [10], and is the precise reason why we would like to advocate this method. For a more recent text on the method, see Bedeaux et al. [15].

To be able to recognize these differences is important, because it enables us to compare energies of systems that vary in size. Consider two nanoparticles at ambient conditions. One is slightly larger than the other, but they are otherwise identical. The particles do not have bare surfaces; molecules will be adsorbed from the environment—for instance, adsorption of CO${}_{2}$ on the surfaces of the particles (not adsorption of nano-sized particles on a macroscopic surface). A central question is then: how do the thermodynamic properties of the adsorbed phase differ between the two cases? This is an important question because the adsorbed phase is an interface through which the particle interacts with its environment. Some forms of energy transfer and some forms of interactions are strongly influenced by the presence of adsorbed phases. Not only would size affect the intensive properties, but depending on the constraints on the system, intensive properties may suffice to determine the system size. At length scales where properties typically are size- and shape-dependent, the nanothermodynamics of Hill may help us investigate the interplay between the nature of a system and its size. This is not to say that other approaches may not be successful (see, e.g., [3]), but the corresponding overarching relations (e.g., Maxwell relations) may be less obvious in these. Therefore, the existence of an alternative method may be useful per se.

Our aim was, therefore, to establish operational thermodynamic relations that enable investigations of thin film properties from a nanothermodynamic perspective. Our work has its basis in the work of Hill on nanothermodynamics [10] and papers V [16] and IX [13] in the series on statistical mechanics of adsorption. The relations were applied to molecular simulations of CO${}_{2}$ adsorption on a spherical graphite-like adsorbent, and to CO${}_{2}$ adsorption on a generic adsorbent with a strong interaction potential. The purpose of the generic adsorbent case was to observe size effects that were thought to possibly occur when there is significant adsorption on very small particles. The outcome was meant to give a foundation for future, similar developments. The contribution of this work is to clarify and extend the work of Hill on nanothermodynamics and provide a new application.

This paper is organized as follows: In Section 2 we derive general thermodynamic relations for a single-component adsorbent with a single-component adsorbed phase. As the application of Hill’s nanothermodynamics is rather limited so far, we considered it necessary to recapitulate the central hypothesis and basis. Readers that are mainly interested in the operational relations and their application may skip this section. Readers that are interested in a comprehensive description of the theory and philosophy of nanothermodynamics are referred to the original work [17] or the recent work [15].

In Section 3 we use the relations to derive operational equations that in turn are applied to the chosen cases. We make simplifying assumptions for the adsorbent, select a reference state typical of adsorption thermodynamics and introduce the condition of equilibrium between the film and the gas. We also derive relations for the dependence of intensive properties on size.

In Section 4 we describe the details of the simulation setup and the methodology for the simulations and the thermodynamic analysis. In Section 5 and Section 6 we present and discuss the simulation results, demonstrating the size dependence of a few select thermodynamic properties. We compare the integral free energy per unit area to the differential change in free energy with respect to the change in area. These are properties that are equal in the macroscopic limit. We compare the enthalpy per molecule to the entropy per molecule times temperature, which are also properties that are equal in the macroscopic limit. Finally, in Section 7 we make concluding remarks and propose directions for future work.

## 2. Nanothermodynamic Framework

In this section, we explain the basic idea of Hill, after considering a common example that motivates his approach; see also Bedeaux et al. [15].

### 2.1. Small vs. Large Systems

Consider a macroscopic, non-volatile adsorbent of one component with adsorbed adsorbate of one component on the surface, in complete equilibrium with a macroscopic gas adsorbate of the same component. The gas is at temperature *T* and chemical potential μ, and the external pressure is completely determined by these two variables. The *adsorbent* may then be taken as a thermodynamic system with a fixed number $N_{A}$ of its component species, in the constant temperature and pressure environment provided by the gas. A large system has the characteristic Gibbs energy $G_{A}\left(T,p,N_{A}\right)=N_{A}f\left(T,p\right)$. Here *f* is a function of *T* and *p*, but independent of the size $N_{A}$ and the amount of adsorbed adsorbate *N*. This implies that contributions of the surface and contributions of the adsorbed adsorbate to the thermodynamic functions of the adsorbent are negligible and that the energy $G_{A}$ is extensive in $N_{A}$. From standard thermodynamics we then have

(1) $$\mu_{A}^{\prime }={\left(\frac{\partial G_{A}}{\partial N_{A}}\right)}_{T,p}=f\left(T,p\right)=\frac{G_{A}}{N_{A}}$$

where the chemical potential is denoted by a mark to ensure it is not confused with the chemical potential in the general Section 2.

Now consider instead the same system, not large, but small enough for the intensive properties to depend on the size, as measured by the value of $N_{A}$. The energy needs to include additional terms related to the size.

The first equality in Equation (1) is still true, but the chemical potential $\mu_{A}^{\prime }$ is now a different function that depends on the size of the system. Furthermore, $G_{A}$ is no longer extensive in $N_{A}$; thus, the relation $\mu_{A}^{\prime }=G_{A}/N_{A}$ is no longer true. The relation is re-established in the macroscopic limit, when the terms related to the system size become insignificant. An exact relation, in place of Equation (1), that is valid for the small system may be given using either Gibbs’ or Hill’s approach. In Hill’s approach a new intensive property denoted by the “hat” symbol is introduced to denote the last member of Equation (1); see [17] (p. 1). Here the property is the new chemical potential ${\hat{\mu }}_{A}^{\prime }\equiv G_{A}/N_{A}$, but for other environments analogous properties are defined. The introduction of this property helps distinguish between the terms $\mu_{A}^{\prime }N_{A}$ and ${\hat{\mu }}_{A}^{\prime }N_{A}$ that appear in the integrated forms of the thermodynamic potentials of the macroscopic and small systems, respectively. That these terms are different is a consequence of the fact that the fundamental equations for small systems are not Euler homogeneous. This is the essential difference between macroscopic and small system thermodynamics. Both $\mu_{A}^{\prime }$ and ${\hat{\mu }}_{A}^{\prime }$ for small systems are functions of $N_{A}$ in addition to *T* and *p*, and are therefore different from the macroscopic chemical potential. In the macroscopic limit $\mu_{A}^{\prime }$ and ${\hat{\mu }}_{A}^{\prime }$ both become equal to the macroscopic chemical potential.

When the environment variables include multiple extensive variables, e.g., *T*, *p*, $N_{1}$, $N_{2}$, …, new intensive properties conjugate to the extensive variables are not defined. Instead, the energy term is referred to as *X*, and depends on the environment. This notation still distinguishes it from the respective terms, e.g., $X\neq \mu_{1}N_{1}+\mu_{2}N_{2}+\ldots$, in the integrated form of the thermodynamic potential for the macroscopic system, but it does not indicate what type of energy it is per se.

### 2.2. Hill’s Extension

The example above shows how properties of small systems may depend on the system size. A relevant theory must therefore allow for variations in size. The theory needs, furthermore, to produce thermodynamic functions and relationships for a single small system. Hill was able to extend large scale thermodynamics to include small systems. In the macroscopic limit his theory becomes identical to the standard thermodynamic equations.

Macroscopic thermodynamics can be applied to a large sample of small systems, such as a macromolecular solution where the small system is the solute macromolecule. In this description we may change the number of small systems, but we cannot change the size of the small system itself. To have a solid foundation for the theory and at the same time allow for variations in size, Hill used the macroscopic thermodynamics of an ensemble of independent small systems as his starting point and introduced size-determining properties as variable parameters. An ensemble is a large collection of systems, where each system replicates the thermodynamic state and environment of the actual thermodynamic system [18]. A member of the ensemble may thus be referred to as a replica.

The system of practical interest in this work is an adsorbent consisting of a single component of quantity $N_{A}$ with an adsorbed phase consisting of a single component of quantity *N*. The system is at temperature *T*, pressure *p* and adsorbed component chemical potential μ, and is completely characterized by *T*, *p*, μ and $N_{A}$. A real system of this type will always exist in the presence of adsorbate molecules—for instance, as free molecules in a gas. The distinction between adsorbed and free molecules is then somewhat arbitrary. There is nothing within the framework of thermodynamics that provides a unique definition of the distinction, so this information must come from elsewhere, such as from experiment or from a theoretical model. In order to stay as general as possible, we make no assumptions in this regard for the time being; cf. Section 4.

In the present case, the method of Hill considers an ensemble of N independent small systems at temperature *T*, pressure *p* and adsorbed component chemical potential μ. All systems have the same assigned amount of adsorbent component $N_{A}$. The ensemble, with total properties denoted by subscript *t*, can now be characterized by the entropy $S_{t}$, volume $V_{t}$, amount of adsorbed component $N_{t}$ and amount of adsorbent component $NN_{A}$. We allow for an independent variation in the number of small systems N and also in the size of the small systems, as given by $N_{A}$. The characteristic function for the ensemble in terms of the set of independent variables $S_{t}$, $V_{t}$, $N_{t}$, $N_{A}$, N is the internal energy $U_{t}$ given by

(2) $$dU_{t}=T\;dS_{t}-p\;dV_{t}+\mu \;dN_{t}+\mu_{A}N\;dN_{A}+X\;dN$$

where *X*, the replica energy, may from Equation (2) be formally defined by

(3) $$X\equiv {\left(\frac{\partial U_{t}}{\partial N}\right)}_{S_{t},V_{t},N_{t},N_{A}}$$

The function *X* describes how a change dN in the number of small systems will change the internal energy of the entire ensemble through XdN. From a physical perspective XdN refers to the process of adding systems to the ensemble, each with the same amount of adsorbent component $N_{A}$, while keeping $S_{t}=NS$, $V_{t}=N\bar{V}$, and $N_{t}=N\bar{N}$ constant. This process explains why *X* may be referred to as the replica energy. For the distribution of small systems over the possible states, this means that the entropy *S*, the mean volume $\bar{V}$ and the mean amount of adsorbed component $\bar{N}$ must decrease. In the process, the ensemble gains the amount $N_{A}\;dN$ of adsorbent component, as opposed to the amount $NdN_{A}$, while the properties $S_{t}$, $V_{t}$ and $N_{t}$ must be redistributed across the new number of systems N+dN. The term XdN is of the same type as the chemical potential term $\mu_{A}N\;dN_{A}$. However, $\mu_{A}$ refers to the addition of a differential amount $dN_{A}$ of adsorbent component for each replica, $N\;dN_{A}$ in total, while *X* refers to the addition of a single system, or an integral amount of adsorbent component $N_{A}$. We see this also by looking at the last two terms in Equation (2) which may be rewritten as $\mu_{A}N\;dN_{A}+X\;dN=\mu_{A}\;d\left(NN_{A}\right)+\left(X-\mu_{A}N_{A}\right)\;dN=\mu_{A}\;dN_{t,A}+\left(X-\mu_{A}N_{A}\right)\;dN$. In the macroscopic limit the ensemble energy is completely characterized by $S_{t}$, $V_{t}$, $N_{t}$ and $N_{t,A}$ and the term $(X-\mu_{A}N_{A})\;dN$ is zero. It follows that in the macroscopic limit $X\to \mu_{A}N_{A}$. This motivates the definition of the new function ${\hat{\mu }}_{A}$ by ${\hat{\mu }}_{A}N_{A}\equiv X$ such that ${\hat{\mu }}_{A}\to \mu_{A}$ in the macroscopic limit. Both ${\hat{\mu }}_{A}$ and $\mu_{A}$ differ from the macroscopic chemical potential at the same *T* and *p*.

The small system can therefore be said to differ from a large one, by the thermodynamic function ε, where $\varepsilon \equiv \left({\hat{\mu }}_{A}-\mu_{A}\right)N_{A}$. This function, which can be viewed as a correction from macroscopic thermodynamics, has been called the subdivision potential, because the process of subdividing the ensemble into a larger number of smaller systems, while keeping $S_{t}$, $V_{t}$, $N_{t}$ and $N_{t,A}$ constant, requires the energy εdN. It has recently been shown to contain interesting size-scaling laws [15,19,20]. The replica energy, the subdivision potential and the scaling laws are particular for the variables that control the ensemble. The presented results refer to an osmotic ensemble, with environment variables *T*, *p*, μ and $N_{A}$.

Next come the thermodynamic functions that follow from Equation (2). By integrating Equation (2) at constant $T,p,\mu ,N_{A}$; defining $U_{t}\equiv N\bar{U}$, $S_{t}\equiv NS$, $V_{t}\equiv N\bar{V}$ and $N_{t}\equiv N\bar{N}$; and dividing by N we obtain the mean internal energy of a single small system given by

(4) $$\bar{U}=TS-p\bar{V}+\mu \bar{N}+{\hat{\mu }}_{A}N_{A}$$

By substituting the definitions for $\bar{U}$, *S*, $\bar{V}$ and $\bar{N}$ in Equation (2) and eliminating ${\hat{\mu }}_{A}N_{A}$ by Equation (4), it follows that

(5) $$d\bar{U}=T\;dS-p\;d\bar{V}+\mu \;d\bar{N}+\mu_{A}\;dN_{A}$$

The differential change in internal energy for a single small system given by Equation (5) has the same form as for a macroscopic system, with the important distinction that the intensive properties are functions of the size of the system. The energy $\bar{U}$ is therefore not a linear homogeneous function of *S*, $\bar{V}$, $\bar{N}$ and $N_{A}$. Therefore we can not obtain Equation (4) by Euler integration of Equation (5). This can be seen clearly if we rewrite Equation (4) as

(6) $$\bar{U}=TS-p\bar{V}+\mu \bar{N}+\mu_{A}N_{A}+\varepsilon$$

where we have used the previous definition $\varepsilon \equiv \left({\hat{\mu }}_{A}-\mu_{A}\right)N_{A}$. The equation is the same as we would expect for a macroscopic system except for the extra term ε. This general feature of small systems has important implications for the Clausius–Clapeyron type equation (Equation (36)), and for the analogue for a small system of the Gibbs adsorption isotherm; see the discussion below in relation to Equation (48). By differentiating Equation (4) and subtracting Equation (5), we obtain

(7) $$d\left({\hat{\mu }}_{A}N_{A}\right)=-S\;dT+\bar{V}\;dp-\bar{N}\;d\mu +\mu_{A}\;dN_{A}$$

This is the function characteristic of a single system of the ensemble for the independent variables $T,p,\mu ,N_{A}$. It is of particular interest operationally because the independent variables are the environment variables. By subtracting $d(\mu_{A}N_{A})$ from Equation (7) we have

(8) $$d\varepsilon =-S\;dT+\bar{V}\;dp-\bar{N}\;d\mu -N_{A}\;d\mu_{A}$$

This equation shows that a small system has one additional independent variable compared to a macroscopic system. In the macroscopic limit, ε=0 and Equation (8) becomes the Gibbs–Duhem equation, a relation between the intensive variables *T*, *p*, μ and $\mu_{A}$ such that only three of them are independent. From Equations (7) and (8) it follows that

(9) $$d{\hat{\mu }}_{A}=-\frac{S}{N_{A}}\;dT+\frac{\bar{V}}{N_{A}}\;dp-\frac{\bar{N}}{N_{A}}\;d\mu -\frac{\varepsilon }{N_{A}^{2}}\;dN_{A}$$

(10) $$d\mu_{A}=-{\left(\frac{\partial S}{\partial N_{A}}\right)}_{T,p,\mu }\;dT+{\left(\frac{\partial \bar{V}}{\partial N_{A}}\right)}_{T,p,\mu }\;dp-{\left(\frac{\partial \bar{N}}{\partial N_{A}}\right)}_{T,p,\mu }\;d\mu -\frac{1}{N_{A}}{\left(\frac{\partial \varepsilon }{\partial N_{A}}\right)}_{T,p,\mu }\;dN_{A}$$

The expression for $d\mu_{A}$ is obtained by considering $\mu_{A}$ a function of *T*, *p*, μ and $N_{A}$, and writing the general expression for the differential. The first three differential coefficients, i.e., in *T*, *p* and μ are obtained by Maxwell relations from Equation (7). By substituting the general expression for $d\mu_{A}$ in Equation (8) and setting *T*, *p*, μ constant, we may solve for the last differential coefficient. Equations (9) and (10) show the integral-differential relation between ${\hat{\mu }}_{A}$ and $\mu_{A}$.

We define the Gibbs energy *G* and the enthalpy *H* by

(11) $$\bar{G}\equiv \bar{U}-TS+p\bar{V}=\mu \bar{N}+\mu_{A}N_{A}+\varepsilon =\mu \bar{N}+{\hat{\mu }}_{A}N_{A}$$

(12) $$\bar{H}\equiv \bar{G}+TS=TS+\mu \bar{N}+{\hat{\mu }}_{A}N_{A}$$

This concludes the theoretical basis needed to describe adsorption on a small adsorbent. We proceed to make the relations operational.

## 3. Operational Relations

The thermodynamic system was defined above as the adsorbent plus the adsorbed film. We are interested in the properties of the film, and how they vary with the size of the adsorbent. The aim is to be able to plot adsorption isotherms, spreading pressure and corresponding film entropy and enthalpy.

### 3.1. The Reference State

As is the usual procedure in adsorption thermodynamics, we define the reference state as the quantity, $N_{A}$, of pure adsorbent, with volume ${\bar{V}}_{0A}$ at external pressure, *p*, and temperature *T*. By pure adsorbent we mean the adsorbent with a clean surface in the absence of adsorption, N→0. The quantities *T*, *p* and $N_{A}$ were defined above; see Section 2. The properties of the film can then be defined as the properties of the total system relative to the reference. In practice, we subtract properties of the pure adsorbent from the total system, while keeping the interaction energy with the film molecules. The equivalents of Equations (4), (5) and (7) for the reference are given by

(13) $${\bar{U}}_{0A}=TS_{0A}-p{\bar{V}}_{0A}+{\hat{\mu }}_{0A}N_{A}$$

(14) $$d\bar{U}=T\;dS_{0A}-p\;d{\bar{V}}_{0A}+\mu_{0A}\;dN_{A}$$

(15) $$d\left({\hat{\mu }}_{0A}N_{A}\right)=-S_{0A}\;dT+{\bar{V}}_{0A}\;dp+\mu_{0A}\;dN_{A}$$

By subtracting Equation (13) from Equation (4), Equation (14) from Equation (5) and Equation (15) from Equation (7), we obtain the equations for the film.

(16) $${\bar{U}}_{s}=TS_{s}-p{\bar{V}}_{s}+\mu {\bar{N}}_{s}-\hat{\Phi }N_{A}$$

(17) $$d{\bar{U}}_{s}=T\;dS_{s}-p\;d{\bar{V}}_{s}+\mu \;d\bar{N}-\Phi \;dN_{A}$$

(18) $$d\left(\hat{\Phi }N_{A}\right)=S_{s}\;dT-{\bar{V}}_{s}\;dp+{\bar{N}}_{s}\;d\mu +\Phi \;dN_{A}$$

where Equation (18) is the characteristic equation for the film in the given environment. Superscript *F* denotes the film properties defined by $S_{s}\equiv S-S_{0A}$, ${\bar{V}}_{s}\equiv \bar{V}-{\bar{V}}_{0A}$, ${\bar{N}}_{s}\equiv \bar{N}$, $\hat{\Phi }\equiv {\hat{\mu }}_{0A}-{\hat{\mu }}_{A}$ and $\Phi \equiv \mu_{0A}-\mu_{A}$. The property ${\bar{N}}_{s}$ is defined for consistency in notation because $\bar{N}$ is already the amount of adsorbed component only.

### 3.2. Size-Dependent Thermodynamic Properties

The analysis continues from here under the approximations that the adsorbent is unaffected by the adsorption, and that its volume, shape and structure are independent of temperature and pressure. The adsorbent then functions only as an external field acting on the adsorbed phase. A more general approach is required for adsorbents that evaporate/dissolve; adsorbents whose structure is affected by the adsorption; and cases where there is no unambiguous definition of the adsorbent’s surface area [13]. However, for the calculations done here, we do not require that level of generality. In general the adsorbent volume may be considered to be a function of *T*, *p* and $N_{A}$. However, under the current approximations the adsorbent thermal expansion and compressibility are negligible. It follows that the adsorbent volume and surface area are functions of $N_{A}$ only. For a constant spherical shape, there is now only one independent variable among the adsorbent volume, surface area and $N_{A}$. The adsorbent surface area Ω is a natural choice when we want to describe the properties of the film only. Eliminating $N_{A}$ in Equations (17) and (18) by substituting $dN_{A}=\left(dN_{A}/d\Omega \right)\;d\Omega$, we have

(19) $$d{\bar{U}}_{s}=T\;dS_{s}-p\;d{\bar{V}}_{s}+\mu \;d\bar{N}-\varphi \;d\Omega$$

(20) $$d\left(\hat{\varphi }\Omega \right)=S_{s}\;dT-{\bar{V}}_{s}\;dp+{\bar{N}}_{s}\;d\mu +\varphi \;d\Omega$$

where we have defined $\varphi \equiv \Phi (dN_{A}/d\Omega )$ and $\hat{\varphi }\equiv \hat{\Phi }\left(N_{A}/\Omega \right)$. The property φ is the usual spreading pressure in adsorption thermodynamics [12]. The property $\hat{\varphi }$ is related to the subdivision potential $\varepsilon_{s}$ by $\varepsilon_{s}=\left(\varphi -\hat{\varphi }\right)\Omega$. The relation can be taken as a definition of the subdivision potential. The equivalents of Equations (9) and (10) are

(21) $$d\hat{\varphi }=\frac{S_{s}}{\Omega }\;dT-\frac{{\bar{V}}_{s}}{\Omega }\;dp+\frac{{\bar{N}}_{s}}{\Omega }\;d\mu +\frac{\varepsilon_{s}}{\Omega^{2}}\;d\Omega$$

(22) $$d\varphi ={\left(\frac{\partial S_{s}}{\partial \Omega }\right)}_{T,p,\mu }\;dT-{\left(\frac{\partial {\bar{V}}_{s}}{\partial \Omega }\right)}_{T,p,\mu }\;dp+{\left(\frac{\partial {\bar{N}}_{s}}{\partial \Omega }\right)}_{T,p,\mu }\;d\mu +\frac{1}{\Omega }{\left(\frac{\partial \varepsilon_{s}}{\partial \Omega }\right)}_{T,p,\mu }\;d\Omega$$

Due to the integral forms of the coefficients in Equation (21) and the differential forms of the corresponding coefficients in Equation (22), we refer to $\hat{\varphi }$ as the integral spreading pressure and to φ as the differential spreading pressure. For Equations (11) and (12) relative to the reference, we see that

(23) $$\bar{G}-{\bar{G}}_{0A}=\mu {\bar{N}}_{s}-\hat{\varphi }\Omega =\mu {\bar{N}}_{s}-\varphi \Omega +\varepsilon_{s}$$

(24) $$\bar{H}-{\bar{H}}_{0A}=TS_{s}+\mu {\bar{N}}_{s}-\hat{\varphi }\Omega =TS_{s}+\mu {\bar{N}}_{s}-\varphi \Omega +\varepsilon_{s}$$

Following the usual procedure in adsorption thermodynamics, the term φΩ is recognized as the analogue of pV for ordinary three-dimensional thermodynamics. We therefore define the film functions ${\bar{G}}_{s}$ and ${\bar{H}}_{s}$ by

(25) $${\bar{G}}_{s}\equiv \bar{G}-{\bar{G}}_{0A}+\varphi \Omega =\mu {\bar{N}}_{s}+\varepsilon_{s}$$

(26) $${\bar{H}}_{s}\equiv {\bar{G}}_{s}+TS_{s}=TS_{s}+\mu {\bar{N}}_{s}+\varepsilon_{s}$$

This ensures the relation between ${\bar{H}}_{s}$ and $S_{s}$ given in Equation (39) when the system is in equilibrium with the gas. The relation in Equation (39) becomes the one usually encountered in adsorption thermodynamics when the system is macroscopic and $\varepsilon_{s}=0$. By Equations (21) and (22) and the relation $\varepsilon_{s}=\left(\varphi -\hat{\varphi }\right)\Omega$ we may write $d\varepsilon_{s}$ in terms of the environment variables T,p,μ,Ω as

(27) $$d\varepsilon_{s}=\Omega^{2}{\left(\frac{\partial S_{s}/\Omega }{\partial \Omega }\right)}_{T,p,\mu }\;dT-\Omega^{2}{\left(\frac{\partial {\bar{V}}_{s}/\Omega }{\partial \Omega }\right)}_{T,p,\mu }\;dp+\Omega^{2}{\left(\frac{\partial {\bar{N}}_{s}/\Omega }{\partial \Omega }\right)}_{T,p,\mu }\;d\mu +\Omega {\left(\frac{\partial \varphi }{\partial \Omega }\right)}_{T,p,\mu }\;d\Omega$$

The differential coefficients of the type ∂(Y/Ω)/∂Ω in Equation (27), where $Y=S_{s},{\bar{V}}_{s},{\bar{N}}_{s}$, may be expanded as

(28) $${\left(\frac{\partial Y/\Omega }{\partial \Omega }\right)}_{T,p,\mu }=\frac{1}{\Omega }\left[{\left(\frac{\partial Y}{\partial \Omega }\right)}_{T,p,\mu }-\frac{Y}{\Omega }\right]$$

This emphasizes the distinction between differential and integral quantities for small systems, one of the key points of nanothermodynamics. In the macroscopic limit, linear homogeneous relations of the type ∂Y/∂Ω=Y/Ω are again true and the bracket term vanishes. It follows directly from Equation (27) that the effects of size on the intensive variables $S_{s}/\Omega$, ${\bar{V}}_{s}/\Omega$, ${\bar{N}}_{s}/\Omega$ and φ may be related to $\varepsilon_{s}$ and changes in $\varepsilon_{s}$ by

(29) $${\left(\frac{\partial S_{s}/\Omega }{\partial \Omega }\right)}_{T,p,\mu }=\frac{1}{\Omega^{2}}{\left(\frac{\partial \varepsilon_{s}}{\partial T}\right)}_{p,\mu ,\Omega }$$

(30) $${\left(\frac{\partial {\bar{V}}_{s}/\Omega }{\partial \Omega }\right)}_{T,p,\mu }=-\frac{1}{\Omega^{2}}{\left(\frac{\partial \varepsilon_{s}}{\partial p}\right)}_{T,\mu ,\Omega }$$

(31) $${\left(\frac{\partial {\bar{N}}_{s}/\Omega }{\partial \Omega }\right)}_{T,p,\mu }=\frac{1}{\Omega^{2}}{\left(\frac{\partial \varepsilon_{s}}{\partial \mu }\right)}_{T,p,\Omega }$$

(32) $${\left(\frac{\partial \varphi }{\partial \Omega }\right)}_{T,p,\mu }=\frac{1}{\Omega }{\left(\frac{\partial \varepsilon_{s}}{\partial \Omega }\right)}_{T,p,\mu }$$

From Equation (21) we have

(33) $${\left(\frac{\partial \hat{\varphi }}{\partial \Omega }\right)}_{T,p,\mu }=\frac{\varepsilon_{s}}{\Omega^{2}}$$

These are the overarching relations inherent in Hill’s formalism that we next may take advantage of in descriptions of further properties of the film. They show the central role of the subdivision potential, which can serve as a direct measure of the system smallness, as we shall see below, e.g., in Equation (37). The relations are not directly obtainable in Gibbs’ treatment of adsorption. From Equation (26) using Equations (29) and (31) it follows that

(34) $${\left(\frac{\partial H_{s}/\Omega }{\partial \Omega }\right)}_{T,p,\mu }=\frac{T}{\Omega^{2}}{\left(\frac{\partial \varepsilon_{s}}{\partial T}\right)}_{p,\mu ,\Omega }+\frac{\mu }{\Omega^{2}}{\left(\frac{\partial \varepsilon_{s}}{\partial \mu }\right)}_{T,p,\Omega }+{\left(\frac{\partial \varepsilon_{s}/\Omega }{\partial \Omega }\right)}_{T,p,\mu }$$

We now consider the special case where the system is in equilibrium with a macroscopic gas at *T*, *p*. The chemical potential μ is then equal to the gas chemical potential $\mu_{G}$. It follows that μ is completely determined by *T* and *p* and that a change dμ is given by $d\mu =-s_{G}\;dT+v_{G}\;dp$, where $s_{G}$ and $v_{G}$ are the entropy and volume per gas molecule. Equation (21) becomes

(35) $$d\hat{\varphi }=\left(s_{s}-s_{G}\right){\bar{\Gamma }}_{s}\;dT-\left({\bar{v}}_{s}-v_{G}\right){\bar{\Gamma }}_{s}\;dp+\varepsilon_{s}/\Omega^{2}\;d\Omega$$

where $s_{s}\equiv S_{s}/{\bar{N}}_{s}$, ${\bar{v}}_{s}\equiv {\bar{V}}_{s}/{\bar{N}}_{s}$, and ${\bar{\Gamma }}_{s}\equiv {\bar{N}}_{s}/\Omega$. It follows that the entropy per film molecule relative to the entropy per gas molecule at the same conditions is given by

(36) $$s_{s}-s_{G}=\left({\bar{v}}_{s}-v_{G}\right){\left(\frac{\partial p}{\partial T}\right)}_{\hat{\varphi },\Omega }$$

This equation together with Equation (46) is applied below to calculate the entropy per film molecule relative to the gas. The relation between the chemical potential of the film and the enthalpy follows from Equation (26), and is given by

(37) $$\mu ={\bar{h}}_{s}-Ts_{s}-\varepsilon_{s}/{\bar{N}}_{s}$$

where ${\bar{h}}_{s}={\bar{H}}_{s}/{\bar{N}}_{s}$. From the equilibrium condition $\mu =\mu_{G}$ it follows that

(38) $${\bar{h}}_{s}-Ts_{s}-\varepsilon_{s}/{\bar{N}}_{s}=h_{G}-Ts_{G}$$

(39) $${\bar{h}}_{s}-h_{G}=T\left(s_{s}-s_{G}\right)+\varepsilon_{s}/{\bar{N}}_{s}$$

This equation was used to calculate the enthalpy per film molecule relative to the gas in simulations.

We can write the equations that relate the effects of size on intensive variables to $\varepsilon_{s}$ in simpler form. Equation (27) becomes

(40) $$d\varepsilon_{s}=\Omega^{2}{\left[\frac{\partial \left(s_{s}-s_{G}\right){\bar{\Gamma }}_{s}}{\partial \Omega }\right]}_{T,p}\;dT-\Omega^{2}{\left[\frac{\partial \left({\bar{v}}_{s}-v_{G}\right){\bar{\Gamma }}_{s}}{\partial \Omega }\right]}_{T,p}\;dp+\Omega {\left(\frac{\partial \varphi }{\partial \Omega }\right)}_{T,p}\;d\Omega$$

The operational equivalents of Equations (29)–(32), now follow directly from Equation (40):

(41) $${\left[\frac{\partial \left(s_{s}-s_{G}\right){\bar{\Gamma }}_{s}}{\partial \Omega }\right]}_{T,p}=\frac{1}{\Omega^{2}}{\left(\frac{\partial \varepsilon_{s}}{\partial T}\right)}_{p,\Omega }$$

(42) $${\left[\frac{\partial \left({\bar{v}}_{s}-v_{G}\right){\bar{\Gamma }}_{s}}{\partial \Omega }\right]}_{T,p}=-\frac{1}{\Omega^{2}}{\left(\frac{\partial \varepsilon_{s}}{\partial p}\right)}_{T,\Omega }$$

(43) $${\left(\frac{\partial \varphi }{\partial \Omega }\right)}_{T,p}=\frac{1}{\Omega }{\left(\frac{\partial \varepsilon_{s}}{\partial \Omega }\right)}_{T,p}$$

From Equation (35) we have the operational equivalent of Equation (33):

(44) $${\left(\frac{\partial \hat{\varphi }}{\partial \Omega }\right)}_{T,p}=\frac{\varepsilon_{s}}{\Omega^{2}}$$

From Equation (39) using Equation (41), we have the operational equivalent of Equation (34):

(45) $${\left[\frac{\partial \left(h_{s}-h_{G}\right){\bar{\Gamma }}_{s}}{\partial \Omega }\right]}_{T,p}=\frac{T}{\Omega^{2}}{\left(\frac{\partial \varepsilon_{s}}{\partial T}\right)}_{p,\Omega }+{\left(\frac{\partial \varepsilon_{s}/\Omega }{\partial \Omega }\right)}_{T,p}$$

### 3.3. Analogue of the Gibbs Adsorption Isotherm

We can integrate Equation (35) at constant *T* and Ω to obtain $\hat{\varphi }$ if we have the adsorption isotherm for a given adsorbent size. We then have

(46) $$\hat{\varphi }=\int_{0}^{p}{\bar{\Gamma }}_{s}\left(v_{G}-{\bar{v}}_{s}\right)\;dp,\;\left(T,\Omega \mathrm{const}.\right)$$

The choice of reference system used to define the film properties can be motivated by this operational equation. As the gas pressure decreases towards zero, the system approaches the reference system. Therefore, $\hat{\varphi }$ vanishes at the lower integration limit. The role of the subdivision potential is no longer visible in the end formula, Equation (46), but as has been seen above, the property can be regarded as an expression of a certain internal structure that must be obeyed.

In standard thermodynamics the well known Gibbs adsorption isotherm may be obtained from the analogue for a surface phase of the Gibbs–Duhem relation at constant temperature [12]. The Gibbs–Duhem relation may be obtained by a standard procedure involving Euler integration of the expression for the differential change in internal energy. When the pure adsorbent is used as the reference state, and the criterion for the distinction between free and adsorbed adsorbate is such that $V_{s}=0$, the analogue of the Gibbs adsorption isotherm is given by Equation (1002,8) in [12], or Equation (15) in [21]. At equilibrium, assuming the gas is an ideal gas, we have $d\mu =d\mu_{G}=v_{G}\;dp\approx kT\;d\ln p$. It follows that the integrated form of the Gibbs adsorption isotherm is given by Equation (29) in [13], or Equation (19) in [21].

The important point here is that direct Euler integration of Equation (19) is not possible. Therefore, the ensemble procedure is used to obtain the analogue of the Gibbs–Duhem relation for a small system, given by

(47) $$d\varepsilon_{s}=d\left[\left(\varphi -\hat{\varphi }\right)\Omega \right]=-S_{s}\;dT+{\bar{V}}_{s}\;dp-{\bar{N}}_{s}\;d\mu +\Omega \;d\varphi$$

which in integrated form at constant *T* and Ω gives Equation (46). Equation (47) further shows the central role of the subdivision potential. Assuming that the gas is an ideal gas, and that the criterion adopted for the distinction between free and adsorbed adsorbate is such that ${\bar{V}}_{s}=0$, the analogue of the Gibbs adsorption isotherm for a small system follows from Equation (46), and is given by

(48) $$\hat{\varphi }=kT\int_{0}^{p}{\bar{\Gamma }}_{s}\;d\ln p,\;\left(T,\Omega \mathrm{const}.\right)$$

In the macroscopic limit the dependence of the intensive properties on the size of the system becomes negligible, and we have $\hat{\varphi }=\varphi$ in Equation (48).

It is interesting to compare the relation between φ and $\hat{\varphi }$ to the relation between $\mu_{A}^{\prime }$ and ${\hat{\mu }}_{A}^{\prime }$; cf. Section 2. Let the adsorbent on which the film is formed be macroscopic such that the film may be considered flat on a scale that is small compared to the size of the adsorbent, but still large compared to the film thickness. The film is then a thermodynamic system characterized by *T*, *p* and Ω. Suppose the film has the energy $\hat{\varphi }\Omega =f\left(T,p\right)\Omega$. The area Ω is then necessary in order to completely characterize the film, but the nature of the film is independent of it. It follows from this expression and Equation (20), with μ=μ(T,p), that

(49) $$\varphi ={\left(\frac{\partial \hat{\varphi }\Omega }{\partial \Omega }\right)}_{T,p}=f\left(T,p\right)=\hat{\varphi }$$

Now consider instead that the system is not macroscopic. The energy may then be alternatively described by including additional terms related to the size of the system. For instance, suppose that the energy is given by

(50) $$\hat{\varphi }\Omega =f\left(T,p\right)\Omega +g\left(T,p,\Omega \right)$$

We would then have

(51) $$\varphi ={\left(\frac{\partial \hat{\varphi }\Omega }{\partial \Omega }\right)}_{T,p}=f+{\left(\frac{\partial g}{\partial \Omega }\right)}_{T,p}$$

(52) $$\frac{\varepsilon_{s}}{\Omega }=\varphi -\hat{\varphi }={\left(\frac{\partial g}{\partial \Omega }\right)}_{T,p}-\frac{g}{\Omega }$$

We see that while the first equality in Equation (49) still holds, the second one does not. Therefore, in order to obtain generalized thermodynamic equations that are valid for the small system represented by Equation (50) and that become the conventional thermodynamic equations in the macroscopic limit, we must, in addition to the regular spreading pressure φ, define the integral spreading pressure $\hat{\varphi }$. The two functions become equal in the macroscopic limit. Then the energy $\varepsilon_{s}\;dN$ required to subdivide the ensemble into a larger number of smaller systems is negligible. It then follows from Equation (52) that the difference between the differential property ${\left(\partial g/\partial \Omega \right)}_{T,p}$ and integral property g/Ω is negligible.

## 4. Methodology

### 4.1. Simulation Techniques

Molecular simulations were done using the open source software LAMMPS (version 20 August 2019) [22] with the grand canonical Monte Carlo simulation technique. A simulation was run for each thermodynamic state of the film characterized by *T*, *p* and Ω. All simulations were bounded by a cubic simulation box with periodic boundary conditions. The size of the box varied depending on the state, but was fixed for any given state. As an example, for all states of the graphite adsorbent system consistent with T=1.08 and Ω≈82, the side length of the box was approximately 15 in reduced units. The gas pressure and density were sampled sufficiently far away from the adsorbent surface for the gas to obtain bulk properties.

The interaction between the gas molecules was described by the Mie potential [23].

(53) $$u_{{}_{G}}\left(r\right)=B\epsilon_{{}_{G}}\left[{\left(\frac{\sigma_{{}_{G}}}{r}\right)}^{\gamma_{r}}-{\left(\frac{\sigma_{{}_{G}}}{r}\right)}^{\gamma_{a}}\right],\;B=\left(\frac{\gamma_{r}}{\gamma_{r}-\gamma_{a}}\right){\left(\frac{\gamma_{r}}{\gamma_{a}}\right)}^{\left(\frac{\gamma_{a}}{\gamma_{r}-\gamma_{a}}\right)}$$

where $\epsilon_{{}_{G}}$ is the energy parameter of the interaction, $\sigma_{{}_{G}}$ is the length parameter of the interaction, $\gamma_{r}$ is the repulsive exponent and $\gamma_{a}$ is the attractive exponent. The parameters were taken from [24] to represent single site coarse-grained CO${}_{2}$ molecules. For the unit of energy we used the strength of the Mie interaction 361.69 kJ, where *k* is the Boltzmann constant. For the unit of length we used the diameter of the CO${}_{2}$ Mie segment 3.741 Å. For the unit of mass, we used the molecular mass of CO${}_{2}$. We set the parameters as follows: (1) The number of Mie segments $m_{s}=1$, (2) $\sigma_{{}_{G}}=1$ by the definition of units, (3) $\epsilon_{{}_{G}}=1$ by the definition of units, (4) $\gamma_{r}=23.0$, (5) $\gamma_{a}=6.66$ and (6) the potential cut-off distance $r_{c,mie}=4.0$.

The interaction between the adsorbent functioning as an external field and a gas molecule, was represented by a spherical colloid potential located at the box center. The expression for this potential follows by integration of the pairwise interactions between a gas molecule and the adsorbent constituent atoms over the adsorbent volume. The interaction between a gas molecule and the adsorbent constituent atom was given by the standard Lennard–Jones 12-6 potential

(54) $$u\left(r^{\prime }\right)=4\epsilon \left[{\left(\frac{\sigma }{r^{\prime }}\right)}^{12}-{\left(\frac{\sigma }{r^{\prime }}\right)}^{6}\right]$$

where $u(r^{\prime })$ is the interaction energy between an adsorbent atom and a gas molecule, $r^{\prime }$ is the center to center distance between them, ϵ is the energy parameter of the interaction and σ is the length parameter of the interaction. By integrating Equation (54) over the spherical adsorbent, we have

(55) $$U\left(r\right)=\frac{16\pi \epsilon \rho_{A}\sigma^{3}}{3}\left[\frac{\left(15R^{3}r^{6}+63R^{5}r^{4}+45R^{7}r^{2}+5R^{9}\right)\sigma^{9}}{15{\left(r^{2}-R^{2}\right)}^{9}}-\frac{R^{3}\sigma^{3}}{{\left(r^{2}-R^{2}\right)}^{3}}\right],\;r>R$$

where U(r) is the interaction energy between the adsorbent and a gas molecule, *r* is the center to center distance between them, $\rho_{A}$ is the adsorbent number density and *R* is the adsorbent radius. The cut-off distance $r_{c}$ for the potential was defined by the relation $U\left(r_{c}\right)/\min\left(U\right)=5.5\times 10^{-3}$, where min(U) is the potential minimum. The value of $r_{c}$, satisfying the relation, was approximated by $r_{c}=1.23R+3.0$ for each adsorbent radius. We used the conventional definition of the Hamaker constant $A_{12}\equiv \pi^{2}C\rho_{1}\rho_{2}$, where *C* is the coefficient of the dispersion energy $u_{disp}\left(r^{\prime }\right)=-C/{\left(r^{\prime }\right)}^{6}$. For a potential of the type in Equation (54) it follows that $C=4\epsilon_{12}\sigma_{12}^{6}$ and

(56) $$A_{12}=4\pi^{2}\epsilon_{12}\rho_{1}\rho_{2}\sigma_{12}^{6}$$

Using Equation (56), and the mixing rules $\sigma =(\sigma_{{}_{G}}+\sigma_{{}_{A}})/2$ and $\epsilon =\sqrt{\epsilon_{{}_{G}}\epsilon_{{}_{A}}}$, we may calculate $\epsilon \rho_{{}_{A}}\sigma^{3}$ in terms of $\sigma_{{}_{G}}$, $\sigma_{{}_{A}}$, $\epsilon_{{}_{G}}$, and $A_{{}_{A}}$ for use in Equation (55). This assumed the adsorbent had the same density as the bodies for which $A_{{}_{A}}$ was measured. Here $\sigma_{{}_{A}}$ and $\epsilon_{{}_{A}}$ are the length and energy parameters of the dispersion energy of two interacting adsorbent atoms, analogous to σ and ϵ, and $A_{{}_{A}}$ is the Hamaker constant for the interaction between two graphite bodies. We take $\sigma_{{}_{A}}=3.4$ Å for the carbon atom–atom interaction, and $A_{{}_{A}}=4.7\times 10^{-19}$ J for the Hamaker constant [25]. It follows that $\epsilon \rho_{{}_{A}}\sigma^{3}=\left[\sqrt{\epsilon_{{}_{G}}A_{{}_{A}}}/\left(16\pi \right)\right]{\left[\left(\sigma_{{}_{G}}+\sigma_{{}_{A}}\right)/\sigma_{{}_{A}}\right]}^{3}\approx 1.79$ in reduced units for the graphite adsorbent system. For the generic adsorbent system we used $\epsilon \rho_{{}_{A}}\sigma^{3}=11.0$, and the cut-off was set to the fixed value $r_{c}=7$.

In order to use the thermodynamic relations with the simulation data, we need to be able to distinguish between adsorbed and free molecules. We therefore define the location of a spherical mathematical dividing surface at a radial distance *a* from the box center by the criterion U(a)=0. We then define the adsorbent volume by $V_{A}\equiv \left(4/3\right)\pi a^{3}$, and the number of adsorbed molecules by $N\equiv N_{B}-\rho_{{}_{G}}\left(V_{B}-V_{A}\right)$, where $N_{B}$ is the number of adsorbate molecules in the box, $\rho_{{}_{G}}$ is the gas density and $V_{B}$ is the box volume. This is a definition based on the concept of surface excess properties introduced by Gibbs [9]. It was easy to apply to the simulations because we had access to the total quantity of the adsorbing component, the gas density and the volume.

### 4.2. Data Reduction

Plots of the integral spreading pressure $\hat{\varphi }\left(\Omega ;T,p\right)$ were first obtained from the adsorption isotherm simulation data of the type ${\bar{\Gamma }}_{s}\left(p;T,\Omega \right)$, one for each area. The following prescription was used to find the desired properties:

- The isotherms were interpolated as described below and integrated using Equation (46), approximating the gas as ideal such that $v_{G}=kT/p$.
- Each of the resulting functions $\hat{\varphi }\left(p;T,\Omega \right)$, one for each area, were then evaluated at the desired pressure to give the final curve.
- The curve for the differential spreading pressure, φ, was obtained from the functions $\hat{\varphi }\left(p;T,\Omega \right)$ for all areas, from the relation $\varphi =\hat{\varphi }+\Omega {\left(\partial \hat{\varphi }/\partial \Omega \right)}_{T,p}$, which follows from Equation (35) at constant *T*, *p* and the definition $\varepsilon_{s}\equiv \left(\varphi -\hat{\varphi }\right)/\Omega$. The derivative $\Omega {\left(\partial \hat{\varphi }/\partial \Omega \right)}_{T,p}$ was approximated by $\Omega {\left(\Delta \hat{\varphi }/\Delta \Omega \right)}_{T,p}$ which was calculated from two functions $\hat{\varphi }\left(p;T,\Omega_{1}\right)$ and $\hat{\varphi }\left(p;T,\Omega_{2}\right)$ for values of $\Omega_{1}$ and $\Omega_{2}$ not too far apart.
- The curve for $\varepsilon_{s}/\Omega$ was obtained as the difference $\varphi -\hat{\varphi }$.
- The curves for the entropy and enthalpy were obtained by Equations (36) and (39), respectively. This required the use of Equation (46) first, so that we knew $\hat{\varphi }\left(p;T,\Omega \right)$. The derivative term was approximated by -$\left(kT/p_{1}\right){\left(\partial p/\partial T\right)}_{\hat{\varphi },\Omega }\approx -\left(kT/p_{1}\right)\left(\Delta p/\Delta T\right)$ which was calculated from two functions $p(\hat{\varphi };T_{1},\Omega )$ and $p(\hat{\varphi };T_{2},\Omega )$ for values of $T_{1}$ and $T_{2}$ not too far apart. The two temperatures used were $T_{1}=1.080$ and $T_{2}=1.165$. The functions were obtained by inversion of $\hat{\varphi }\left(p;T_{1},\Omega \right)$ and $\hat{\varphi }\left(p;T_{2},\Omega \right)$.

For all state properties of which the approximate value was calculated from two states, i.e., from expressions containing finite difference terms such as $\Omega {\left(\Delta \hat{\varphi }/\Delta \Omega \right)}_{T,p}$, the association of the value with a single state is somewhat arbitrary. If we label the states in the difference above as $\Omega \left(\Delta \hat{\varphi }/\Delta \Omega \right)=\Omega \left({\hat{\varphi }}_{2}-{\hat{\varphi }}_{1}\right)/\left(\Omega_{2}-\Omega_{1}\right)$ we chose to assign the values to the state of ${\hat{\varphi }}_{1},\Omega_{1}$. This means that $\Omega =\Omega_{1}$ in the expression. Another choice may be to assign the value to some mean of the states. In the limit of infinitesimal differences, both choices give the same curves. The choice we made has the advantage of being simpler to implement in software. However, if quantitative accuracy is the main concern, the second choice may be more satisfactory.

In summary we acquired simulation data for a range of pressures and areas at two different temperatures. We set the control parameters *T*, μ and Ω for each simulation and sampled the gas pressure and density from the region of macroscopic gas properties. The total amount of the adsorbate component was also sampled. The rest of the properties then followed by thermodynamic relations. In each simulation the system was first equilibrated for a number of cycles that depended on the state we were simulating. The properties of interest were monitored to make sure they fluctuated around a steady value. This was followed by a number of cycles where samples for calculating ensemble averages and errors were collected. The number of cycles varied with the state simulated, and were chosen in each case such that the estimated error was less than 1.5% of the mean with 95% confidence for all sampled properties and the calculated property ${\bar{\Gamma }}_{s}$. The strongest correlation between samples was found in ${\bar{\Gamma }}_{s}$. This property therefore determined the number of cycles. In estimating the standard error we accounted for correlation in the data by block analysis.

We analyzed the data using the scientific computing library SciPy [26], and created figures using Matplotlib [27]. We interpolated the simulation data for the adsorption isotherms ${\bar{\Gamma }}_{s}\left(p;T,\Omega \right)$ using univariate splines, constraining the splines to be linear for the lowest pressures according to Henry’s law. We then extrapolated the linear region to zero pressure to allow the integration in Equation (46). We evaluated the integral analytically for the lowest pressures, and numerically for the remaining pressures.

## 5. Results

**Figure 1:**
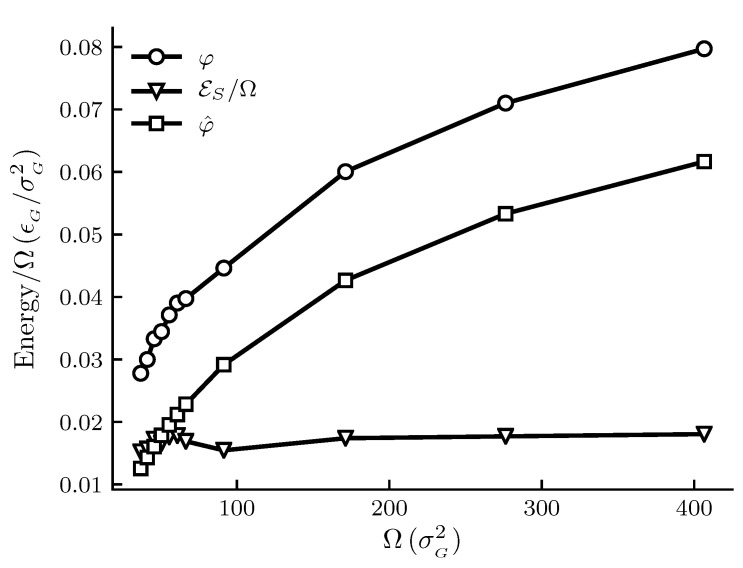
Film properties φ, $\hat{\varphi }$ and $\varepsilon_{s}/\Omega$ for the graphite adsorbent case as functions of the adsorbent area Ω at constant temperature T=1.080 and pressure p=0.011. All quantities are given in reduced units. For reference, the approximate values in SI units are T≈250 K, p≈1.05 MPa and adsorbent radii are between 6 and 25 × 10${}^{-10}$ m. The markers indicate which adsorbent sizes were simulated and the lines are there as visual aid.

**Figure 2:**
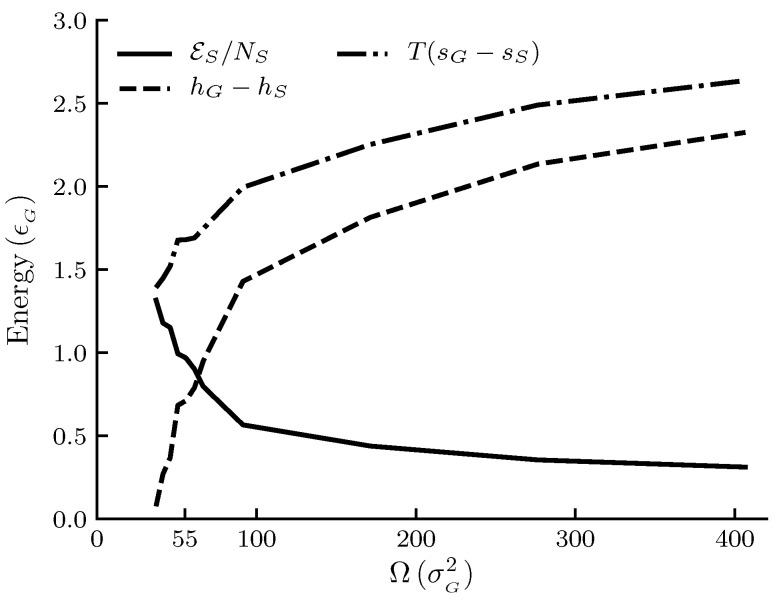
Film properties enthalpy, entropy and the subdivision potential per film molecule as functions of the adsorbent area Ω at constant temperature T=1.080 and pressure p=0.011. Enthalpy and entropy for a film molecule are given relative to the gas. All quantities are given in reduced units. For reference, the approximate values in SI units are T≈250 K, p≈1.05 MPa and adsorbent radii are between 6 and 25 × 10${}^{-10}$ m for the area range.

**Figure 3:**
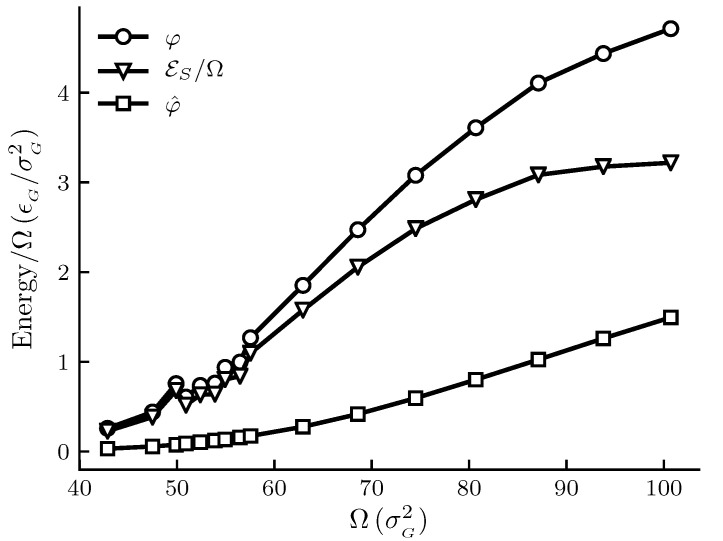
Film properties φ, $\hat{\varphi }$ and $\varepsilon_{s}/\Omega$ as functions of the adsorbent area Ω at constant temperature T=1.080 and pressure *p* = 1.8× 10${}^{-4}$ for the generic adsorbent case. All quantities are given in reduced units. For reference, the approximate values in SI units are T≈250 K, p≈50 kPa and adsorbent radii are between 6 and 9 × 10${}^{-10}$ m. The markers indicate which adsorbent sizes were simulated and the lines are there as a visual aid.

**Figure 4:**
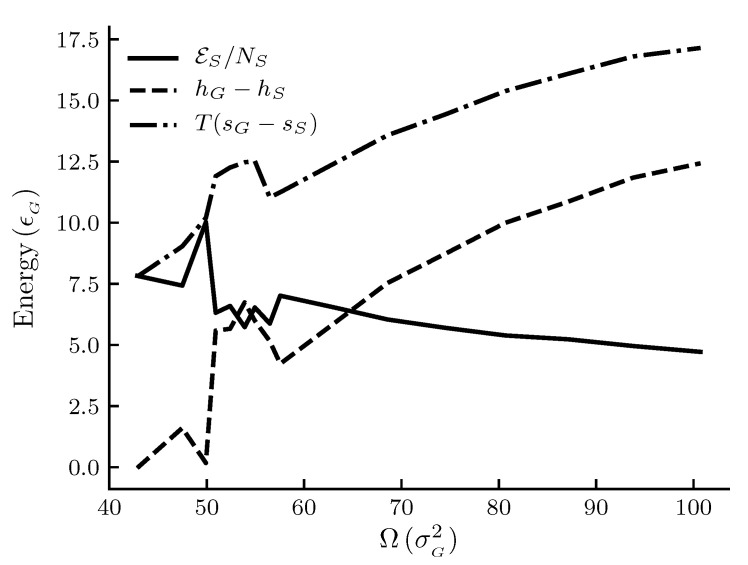
Film properties enthalpy, entropy and the correction function per film molecule as functions of the adsorbent area Ω at constant temperature T=1.080 and pressure *p* = 1.8 × 10${}^{-4}$ for the generic adsorbent case. Enthalpy and entropy for a film molecule are given relative to the gas. All quantities are given in reduced units. For reference, the approximate values in SI units are T≈250 K, p≈50 kPa and adsorbent radii are between 6 and 9 × 10${}^{-10}$ m for the area range.

The calculated results are shown in Figure 1, Figure 2, Figure 3 and Figure 4. The figures show φ, $\hat{\varphi }$ and $\varepsilon_{s}/\Omega$ and other thermodynamic properties as functions of the adsorbent area Ω at constant temperature and pressure. All quantities are given in reduced units. Figure 1 and Figure 2 are the results for the graphite adsorbent case. Figure 3 and Figure 4 are the results for the generic adsorbent case representing very small adsorbents with strong interaction potentials. It is clear from the figures that all properties shown depend on the system size (area). To place our experiment in context with macroscopic thermodynamics, consider an infinite flat film adsorbed on a flat adsorbent. The intensive thermodynamic properties of this film do not depend on the surface area. There is then no difference between the integral spreading pressure $\hat{\varphi }$ and the differential spreading pressure φ, and $\varepsilon_{s}=0$. The results documented show that we are far away from this limit, since there is an observable difference between integral and differential properties. The systems can thus be considered as small in Hill’s sense.

Figure 1 shows how the spreading pressures, φ and $\hat{\varphi }$, and the subdivision potential depend on system size for the graphite adsorbent case. The area range corresponds approximately to an adsorbent radius between 6 and 25 × 10${}^{-10}$ m. We can see that the trend for φ and $\hat{\varphi }$ has an initially steep increase, after which the rate of increase goes down. At the upper limit of the area range there is still a significant difference between φ and $\hat{\varphi }$. The subdivision potential per unit area $\varepsilon_{s}/\Omega$, or the deviation from the corresponding macroscopic system, appears fairly constant; however, these results are still consistent with the theoretical prediction that $\varphi =\hat{\varphi }$ and $\varepsilon_{s}$ approaches zero in the macroscopic limit; see discussion below.

Figure 2 shows the size dependence of the entropy and the enthalpy per film molecule relative to the gas, and the difference $\varepsilon_{s}/{\bar{N}}_{s}$ between the two as given by Equation (39) for the graphite adsorbent case. We see that, in general, there is less entropy and enthalpy per film molecule as the adsorbent becomes larger. The rate at which the properties decrease with increasing size goes down. The difference $\varepsilon_{s}/{\bar{N}}_{s}$, or the deviation from the corresponding macroscopic system, decreases as the adsorbent area becomes larger. This is expected.

Figure 3 shows how the spreading pressures, φ and $\hat{\varphi }$, and the subdivision potential depend on system size for the generic adsorbent case. We see that the general trend for all three curves is an initial increase to an inflection point, after which the rate of increase goes down. At the upper limit of the area range there is still a significant difference between φ and $\hat{\varphi }$, and the subdivision potential per unit area $\varepsilon_{s}/\Omega$ has not started to decrease towards zero at that point. However, the results are still consistent with the theoretical prediction that $\varphi =\hat{\varphi }$ and $\varepsilon_{s}$ approaches zero in the macroscopic limit; see discussion below. Special is a local deviation from the overall trend of the curves for adsorbent areas 50<Ω<60. This is more clearly seen in the enthalpy and entropy curves of Figure 4 for the same range of areas.

Figure 4 shows the size dependence of the entropy and the enthalpy per film molecule relative to the gas, and the difference $\varepsilon_{s}/{\bar{N}}_{s}$ between the two as given by Equation (39). We see that, in general, there is less entropy and enthalpy per film molecule as the adsorbent becomes larger. The rate at which the properties decrease with increasing size goes down. The difference $\varepsilon_{s}/{\bar{N}}_{s}$, or the deviation from the corresponding macroscopic system, decreases as the adsorbent area becomes larger. This is expected.

A local maximum in the entropy of the gas relative to the entropy of the film for adsorbent areas 50<Ω<60 is special, as is a corresponding maximum in the gas enthalpy relative to the film enthalpy. The quantities are plotted as $T(s_{G}-s_{s})$ and $h_{G}-{\bar{h}}_{s}$ for convenience so that all quantities are positive. Thus we observed a minimum in the film entropy relative to the gas $s_{s}-s_{G}$ and a minimum in the film enthalpy relative to the gas ${\bar{h}}_{s}-h_{G}$. From the results, the areas around reduced units 55 appear special. Compared to Figure 2 it appears that there is local deviation from the general trend in the curves for the graphite adsorbent in this range of areas, but to a much smaller extent than for the generic adsorbent. A thermodynamic analysis alone will not reveal any particular reason for such a behavior. Several explanations can be thought of, as discussed below.

## 6. Discussion

In the simulations we changed the size of the adsorbent while keeping the temperature and chemical potential of the gas constant. As the gas is macroscopic, this is equivalent to controlling the temperature and pressure. In effect, as long as the gas is macroscopic it is unaffected by the size of the adsorbent. In cases where the gas is no longer macroscopic, such as in confinement, the gas phase must also be treated as a small system where all three of the variables *T*, *p* and μ are independent.

When we increase the size of the adsorbent, there are two direct changes to the composite system: (1) the strength of the external field increases, and (2) the surface becomes less curved. For the present systems, the two changes were coupled to the size of the adsorbent, so we cannot isolate the individual effects. However, if one would like to do so, one could study different adsorbent materials at the same size.

The strength of the external field felt by an adsorbed molecule comes from integrating the pairwise sum over all atoms that would constitute the adsorbent, resulting in Equation (55). Therefore, an increase in the field strength is analogous to the introduction of more adsorbent atoms. This leads to a larger total interaction energy for the film, but is to some extent counteracted by the fact that with increasing size, a larger fraction of the adsorbent atoms would be at a greater distance from a given adsorbed molecule. As the pair potential is inversely proportional to the separation distance by $u\left(r\right)\propto r^{-6}$, the greater distance leads to a plateau in the total interaction energy in the large size limit.

To further discuss the effect of the presence of the adsorbent and its size on the film, we give an illustration of four related systems divided into two groups. The first group consists of two systems that have flat surfaces, while the second group consists of two systems with curved surfaces. In each group one of the systems has the condensed phase in the presence of an adsorbent while the other has a free condensed phase. The comparison of free and adsorbed systems within a group focuses on the effects of the adsorbent, while the comparison of spherical and flat systems between the groups focuses on the effects of size. We found the analogy of the systems in the second group to the more simple systems in the first group helpful.

In the first case, consider a single-component bulk liquid with a flat surface, in equilibrium with its own vapor. The nature of this system is completely characterized by the temperature. This means that there is only one pressure $p^{0}\left(T\right)$ for a given temperature *T* at which the equilibrium state can exist. If we now extend the definition of the system and let the liquid be adsorbed onto a flat adsorbent surface, the situation becomes different. If the pressure is below $p^{0}$ for the given temperature, the phase will start to evaporate. However, the evaporation will not necessarily continue until there is only gas left. This is because the evaporation will reach a point at which the field of force from the adsorbent is felt in such a way that no part of the phase maintains the properties of bulk. Beyond this point there is a non-zero free energy change associated with adsorption/desorption, and the amount of adsorbed material ${\bar{\Gamma }}_{s}\left(T,p\right)$ may adjust to satisfy the equilibrium condition while *T* and *p* remain independent. The condensed phase may therefore be in equilibrium with the vapor at pressures below $p^{0}$.

Now consider the analogous case of a single-component spherical liquid droplet of area $\Omega^{*}$ in equilibrium with its own vapor. The (unstable) equilibrium state is characterized by *T* and $\Omega^{*}$. This means that for a given temperature and area there is only one pressure $p^{0^{\prime }}>p^{0}$ at which the equilibrium state can exist. The equilibrium pressure $p^{0^{\prime }}\left(T,\Omega^{*}\right)$ for this system is larger than $p^{0}$ due to the curvature and the Laplace pressure. If we let the liquid be an adsorbed phase on a spherical adsorbent, as in our simulations, the situation changes again. The system has three independent variables. The equilibrium state may be characterized by *T*, Ω and *p* as in Equation (21). Compared to the droplet at the same temperature, we set the adsorption such that the two physical systems are equal in size as characterized by $\Omega^{*}$. There is one more independent variable to fix. Therefore, unlike for the droplet we have many possible equilibrium pressures for a given adsorption (as determined by $\Omega^{*}$) if we let Ω be different in each case. In Figure 1, Figure 2, Figure 3 and Figure 4 we have many adsorptions for a given pressure because the area is different in each case. For given values of *T* and Ω there is a limit pressure $p^{0^{\prime }}$ at which bulk condensation starts. This limit becomes lower for larger Ω until it reaches $p^{0}$ for a large flat adsorbent. In other words, a given adsorbent size imposes a limit on the vapor pressure required for bulk condensation. This has previously been observed with a statistical model [28]. As the limit changes with size, this implies that a change in size of the adsorbent for $p^{0}<p<p^{0^{\prime }}$ may induce bulk condensation if $p^{0^{\prime }}$ drops below *p*. For $p<p^{0}$ there is naturally no adsorbent size that will induce bulk condensation. After bulk condensation starts, the saturation pressure decreases gradually towards $p^{0}$ because the curvature of the growing condensed phase decreases and approaches zero.

To summarize, for the first system we only have to fix the temperature in order to fix the equilibrium state. In the second system we have, in addition, to fix the pressure. In the third system we have to fix the temperature and the area of the adsorbent. In the last system we have to fix the temperature, the area and the pressure.

For our simulations the illustration above implies that for pressures $p<p^{0}$, we may have a flat and a spherical adsorbed film in equilibrium at the same temperature and pressure. They will in general have different thermodynamic properties. For instance, the adsorption differs as discussed above. By increasing the adsorbent size, the bulk condensation pressure limit approaches $p^{0}$ from above and the thermodynamic properties approach the values for a flat film. For pressures $p>p^{0}$, the spherical film may be in equilibrium but there is no possible corresponding flat system because $p^{0}<p^{0^{\prime }}$. An increase of adsorbent size may cause the bulk condensation pressure limit $p^{0^{\prime }}$ to pass through *p*, at which point the adsorption diverges.

In Figure 1 and Figure 3 the pressures are below $p^{0}$ for the given temperature. We would therefore expect the curves for φ and $\hat{\varphi }$ to eventually coincide at the plateau value for a flat adsorbed film at the given temperature and pressure. This occurs when the slope of the $\hat{\varphi }$ curve is zero, which is consistent with the relation $\varphi =\hat{\varphi }+\Omega {\left(\partial \hat{\varphi }/\partial \Omega \right)}_{T,p}$ and Equation (44) as $\varepsilon_{s}=0$. The curve for $\hat{\varphi }$ in Figure 3 is s-shaped and consistent with the approach to a plateau value. According to Equation (44), the s-shaped curve of $\hat{\varphi }$ gives a bell-shaped curve for $\varepsilon_{s}/\Omega$, with a peak value at the inflection point of $\hat{\varphi }$, and ending at ε/Ω=0. The expected curve for φ would according to the relation $\varphi =\hat{\varphi }+\Omega {\left(\partial \hat{\varphi }/\partial \Omega \right)}_{T,p}$ also have a peak before decreasing towards the plateau value of $\hat{\varphi }$. The same reasoning applies to Figure 1, however the shapes of the curves are stretched out and harder to identify. Thus, although the eventual decrease in $\varepsilon_{s}$ towards zero is not observed for the limited area ranges in Figure 1 and Figure 3, the results are consistent with the macroscopic limit relations $\varphi =\hat{\varphi }$ and $\varepsilon_{s}=0$. More experiments are needed for further confirmations.

The trends in the entropy and enthalpy curves in Figure 2 and Figure 4 are reasonable, when we consider the discussion above on the change in total interaction energy with size. With a larger interaction energy, the molecules are more tightly bound to the surface, and there are less possible configurations of the system available. The adsorbed phase is then more ordered, and the entropy becomes lower. In the macroscopic limit where the film is flat $\varepsilon_{s}/{\bar{N}}_{s}=0$ and ${\bar{h}}_{s}-h_{G}=T\left(s_{s}-s_{G}\right)$ by Equation (39). The trends of the curves for $\varepsilon_{s}/{\bar{N}}_{s}$ are consistent with this. More experiments are needed for further confirmations.

For the very small adsorbent with a strong interaction potential(cf. Figure 4), we can observe a peak in the entropy and enthalpy, and there is a corresponding effect in Figure 3. A minimum in the entropy of an adsorbed molecule, when plotted against the adsorption or pressure, is well known in systems where the interaction of the adsorbate with the adsorbent is strong. This is related to the decrease in the number of configurations available to the system as the first adsorption layer is filled and a subsequent increase following the initiation of adsorption in a second layer. No film density variation consistent with this was observed. The peak we see in Figure 4 was also observed for the same areas for a range of different adsorptions, indicating that it does not have the same origin as discussed above. These were observations from curves showing the same qualitative behavior but at different pressures than the ones shown here. The start of the second layer was estimated by the location of the minimum in the entropy when plotted against the adsorption (not shown here). The local minimum we observe may thus best be related to available configurations of the system in other ways, such as the change in structure and arrangement of the molecules on the surface. The type of entropy we are showing in Figure 4 is the entropy per molecule $S_{s}/{\bar{N}}_{s}$ (relative to the gas). As emphasized by Hill [16], this type of entropy, as opposed to the differential entropy ${\left(\partial S_{s}/\partial {\bar{N}}_{s}\right)}_{T,\Omega }$ is the correct one to discuss in relation to the degree of order of the adsorbed molecules. More information on the spatial arrangement of the adsorbed molecules may allow this question to be investigated further.

## 7. Conclusions and Perspectives

We have seen in this work how we can deal with size as a variable in a systematic manner. The common thermodynamic equations for adsorption were changed in three ways.

Using the procedure of Hill, we have calculated the integral spreading pressure from adsorption isotherm data for a fixed adsorbent size, using Equation (46). The equation has the same form as the one usually encountered in adsorption thermodynamics, the integral form of Equation (26) in [13], with the important distinction that $\hat{\varphi }$ and φ in Hill’s description are not the same functions for small systems. Equation (46) is valid for systems of any size.

Similarly, we have shown how to obtain the entropy and the enthalpy per film molecule by Equations (36) and (39). Equation (36) is the same as the one usually encountered in adsorption thermodynamics, Equation (21) in [13], except for the important fact that the function that is kept constant when we take the derivative is now $\hat{\varphi }$ instead of φ.

This entropy is the entropy typically discussed in relation to the degree of order of the adsorbed molecules. Equation (39) differs from the usual equation by the term $\varepsilon_{s}/{\bar{N}}_{s}$. We observe that for a small system ${\bar{h}}_{s}-h_{G}\neq T\left(s_{s}-s_{G}\right)$ because $\varepsilon_{s}\neq 0$. The expressions referred to above, become the usual expressions in the macroscopic limit where $\varepsilon_{s}=0$ and $\hat{\varphi }=\varphi$.

The effects of size on intensive variables is a feature of Hill’s nanothermodynamics [17], and may be expressed in terms of $\varepsilon_{s}$ and derivatives of $\varepsilon_{s}$ by Equations (41)–(45). By the subdivision potential, we have a direct measure of the system smallness, and we can see from the numerical data that the value is significant in all relevant properties.

On this basis, we can suggest some possible directions for future work. The adsorbent may be modeled explicitly as a collection of atoms instead of as an integrated potential. This may allow the investigation of adsorption on a non-smooth surface with adsorption sites. By allowing the adsorbent to be compressible, it may be possible to observe how the film is affected by a phase transition in the adsorbent for different sizes. By modeling the adsorbate as all-atom molecules instead of coarse-grained particles, it may be possible to study changes in the molecular orientation and structure of adsorbing layers induced by the adsorbent size.
